# Designing Adverse Event Forms for Real-World Reporting: Participatory Research in Uganda

**DOI:** 10.1371/journal.pone.0032704

**Published:** 2012-03-29

**Authors:** Emma C. Davies, Clare I. R. Chandler, Simeon H. S. Innocent, Charles Kalumuna, Dianne J. Terlouw, David G. Lalloo, Sarah G. Staedke, Ane Haaland

**Affiliations:** 1 Liverpool School of Tropical Medicine, Liverpool, United Kingdom; 2 London School of Hygiene and Tropical Medicine, London, United Kingdom; 3 Cambridge University, Cambridge, United Kingdom; 4 Uganda Malaria Surveillance Project, Kampala, Uganda; 5 University of Oslo/Haaland Communication, Oslo, Norway; Mahidol-Oxford Tropical Medicine Research Unit, Thailand

## Abstract

The wide-scale roll-out of artemisinin combination therapies (ACTs) for the treatment of malaria should be accompanied by continued surveillance of their safety. Post-marketing pharmacovigilance (PV) relies on adverse event (AE) reporting by clinicians, but as a large proportion of treatments are provided by non-clinicians in low-resource settings, the effectiveness of such PV systems is limited. To facilitate reporting, AE forms should be easily completed; however, most are challenging for lower-level health workers and non-clinicians to complete. Through participatory research, we sought to develop user-friendly AE report forms to capture information on events associated with ACTs.

Following situation analysis, we undertook workshops with community medicine distributors and health workers in Jinja, Uganda, to develop a reporting form based on experiences and needs of users, and communication and visual perception principles. Participants gave feedback for revisions of subsequent versions. We then conducted 8 pretesting sessions with 77 potential end users to test and refine passive and active versions of the form.

The development process resulted in a form that included a pictorial storyboard to communicate the rationale for the information needed and facilitate rapport between the reporter and the respondent, and a diary format to record the drug administration and event details in chronological relation to each other. Successive rounds of pretesting used qualitative and quantitative feedback to refine the form, with the final round showing over 80% of the form completed correctly by potential end users.

We developed novel AE report forms that can be used by non-clinicians to capture pharmacovigilance data for anti-malarial drugs. The participatory approach was effective for developing forms that are intuitive for reporters, and motivating for respondents. The forms, or their key components, could be adapted for use in other low-literacy settings to improve quality and quantity of drug safety reports as new medicines are scaled-up.

## Introduction

Since 2004, artemisinin combination therapies (ACTs) have been scaled-up rapidly worldwide. ACTs have a good safety profile in clinical trials and are among some of the most widely used drugs in the world. However, programmatic safety data from the ‘real world’, where drugs will be used repeatedly and often presumptively at health centre and community level in high-transmission settings, cannot be captured well by standard post-marketing pharmacovigilance systems in endemic areas [1]. Post-marketing pharmacovigilance is intended to build on safety data obtained in clinical trials, aiming to collect sufficient quality data to be able to identify rare, serious adverse events (AEs), and to obtain more information on the nature and burden of known AEs in the general population. Despite encouragement for inclusion of pharmacovigilance activities in national malaria control plans [2], implementation has been inconsistent, and pharmacovigilance coverage remains low in many countries [3].

Improved pharmacovigilance requires more data of better quality. However, reporting adverse events in programmatic settings can be particularly challenging, and forms often require individuals completing the forms to negotiate, translate and interpret this complexity. Although a valid AE report requires only four essential fields; patient, reporter, drug name and event description [4]; more detailed information about the patient and the event is required to conduct meaningful assessments and to characterise emerging safety signals with reliability. This complexity is likely to contribute to the low numbers of events reported and the poor quality of reports.

Relatively little attention has been paid to the way information is recorded on reporting forms. Most countries rely primarily on spontaneous adverse drug reaction (ADR) reporting schemes similar to the UK's Yellow Card scheme [5]. However, reporting forms tend to be developed by those interpreting the forms, rather than those collecting the data. They may also assume that the patient's history is captured elsewhere, as would be the case in a controlled-trial setting. Such assumptions, embedded into the design of reporting forms, limit their broader usability and usefulness. Formal and informal health workers, commonly involved in malaria treatment provision in sub-Saharan Africa, may be unfamiliar with formal pathways for reporting, and the complexity of AE forms may further deter them from reporting [6]. Patients may not report due to fear of incrimination from health workers and perception that some AEs are actually indicators of drug efficacy, rather than side-effects, such as itching with chloroquine [6]–[8]. Further complicating reporting of adverse events following antimalarial treatment is the challenge of distinguishing between symptoms of malaria and AEs [9], [10]. Reporting forms that present the course of events clearly, with minimal need for interpretation at the time of recording the event, are therefore essential. Forms suitable for passive and active data collection are also needed. Passive data collection forms are required for spontaneous reporting, when patients present with symptoms that are reported as an AE. Active data collection forms are needed for clinical trials or cohort event monitoring, to monitor all patients in the study population.

We developed new passive and active reporting forms to document AEs following treatment with ACTs, aiming to produce a format that targets issues most important to those involved in reporting, to improve detection of adverse drug reactions and to enhance collection of high quality information by lower-level health workers involved in pharmacovigilance.

## Methods

We used participatory methods to develop a new reporting form. This approach engages the target audience in the problem defining and solving process [11], in order to produce interventions that are more likely to be taken up by end users.

### Project site and team

This project is part of a larger study to collect safety data from studies conducted by the ACT Consortium (www.actconsortium.org) in seven countries. We carried out this work in Uganda, where we were familiar with the pharmacovigilance system, and where AE reporting in communities and at drug shops was ongoing, supported by the Uganda Malaria Surveillance Project (UMSP). Fieldwork took place in Budondo, a rural sub-county in Jinja district. Final pretesting took place in Kampala. The field team consisted of a pharmacist (ED), social scientists (CC and SI), a social scientist/communication specialist (AH), two clinicians (SS and CK), two local artists and a team of 5 social science assistants who were trained for the pretesting activities (by AH) following best practice methods [12]–[14].

### Participants

We selected participants to represent those who would potentially collect AE data. In Budondo, community medicine distributors (CMDs), health workers from public health facilities, and UMSP fieldworkers were selected. CMDs are literate members of the community, without formal medical training, who were elected to participate in Uganda's home-based management of fever (HBMF) programme. Many CMD participants were also involved in the UMSP pharmacovigilance programme and had experience reporting AEs. In Kampala, members of the UMSP research team conducting pharmacovigilance activities and university graduates were recruited to pretest the final versions of the reporting forms. Graduates were selected to represent those likely to be recruited as non-clinical field workers in research projects.

### Conceptual framework

The process of AE report generation typically involves transformation, or ‘concretisation,’ of an individual's experience into a report relevant for pharmacovigilance. First, the patient or caregiver must decide whether their experience merits action contingent upon their understanding of illness and disease, and the perceived ease and outcomes of different actions, including reporting the adverse experience as an event. Second, their subjective illness and treatment experience must be translated into what they consider to be medically recognized categories [7]. Third, the reporter must filter and concretise the experience further into what they consider to be empirically verifiable biomedical phenomena that can be written down and hold meaning for those interpreting the report. Fourth, the reporter or a third party must interpret the respondent's experience, now considered to be ‘data,’ and assign what they consider to be the severity of the event, and causality between the event and treatment received. In each of these stages, cultural expectations, including from ethnomedical and biomedical paradigms, and communicated by the reporter to the respondent, shape what is considered relevant to report. The process is akin to that of the transformation from illness to disease, from a subjective experience to a physical and measurable phenomenon [15].

### Project design

**Figure 1:**
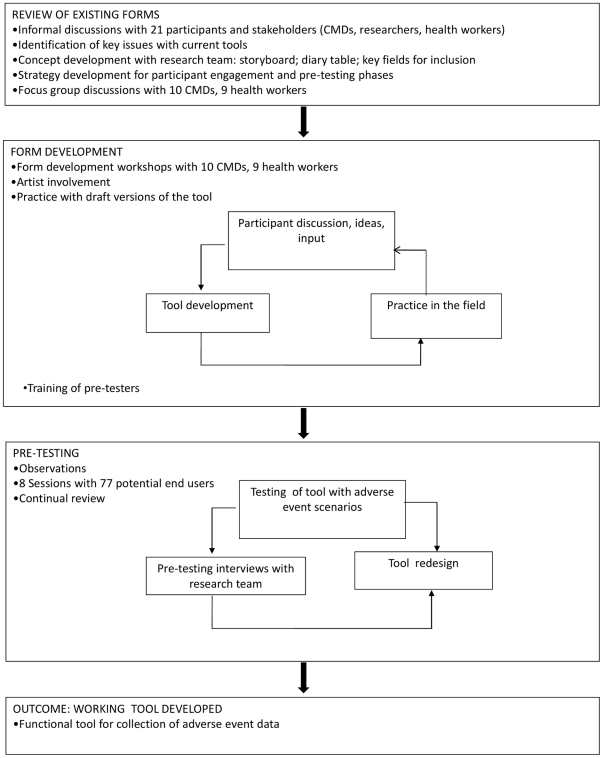
Overview of project design.

The project involved three phases: (1) Review of existing forms, (2) form development, when intended end-users participated in workshops to help design simple forms, and (3) pretesting, which involved one observation phase with CMDs and a formal testing phase with graduate fieldwork recruits and CMDs in Kampala (Figure 1). The fieldwork activities overlapped with analysis, allowing the team to plan the next steps. Analysis of interviews, discussions and observations involved manually reviewing transcripts and fieldnotes for main themes and concerns of participants, drawing out those related to AE reporting. Quantitative analysis of pretesting involved descriptive statistics using Excel.

#### Review of existing forms

To understand how current pharmacovigilance reporting forms were being used in Uganda, we undertook a review of three types of AE forms completed by different health care providers: clinicians, drug-shop owners, and CMDs. The first was the Ugandan National Drug Authority (NDA) form; the others were developed by UMSP for use in pharmacovigilance activities. We noted where problems occurred in previously completed forms and then conducted a field visit to observe and discuss the use of forms by a small convenience sample of health workers, CMDs and drug shop workers active in local pharmacovigilance projects. We discussed these findings with project staff responsible for interpreting AE forms and entering data, to understand their perspectives on the reporting system and ideas for improvements. We then reviewed the forms in two focus group discussions with CMDs and then health workers, in which participants were encouraged to discuss their experiences with anti-malarial treatment and reporting of AEs with existing forms and procedures.

#### Form development

In the form development phase, we produced a draft form for passive reporting. We held two participatory workshops, one with CMDs and one with health workers. In the workshops participants were divided into small groups and asked to draft a simple reporting form, basing the layout on their own understanding of what was important to include. A local artist drafted and refined sketches at the request of participants. The artist was briefed on principles for drawing pictures that are understandable to low-literacy audiences [16].

Participants in the first workshop were then presented with ideas originating from our evaluation of the challenges and opportunities presented by the forms reviewed previously. Participants were invited to consider whether to incorporate these ideas into their own forms. We allowed time to develop trust that we wanted the participants' ideas and contributions in order to establish motivation and commitment towards the development of a good quality form. Facilitators moved between the working groups to encourage participants to include essential data fields for pharmacovigilance within the form. We asked groups to practice using their draft forms in role plays, and to make relevant adaptations.

Following their workshop, CMDs were asked to take the latest draft form home to practice recording AEs, and were invited to attend a follow-up workshop to give feedback on the existing draft.

#### Pretesting

Prior to formal pre-testing, to evaluate how the new reporting forms would work in practice, we asked the CMDs and health workers to practice with the forms informally, using friends and neighbours as informants, with real cases where possible, alongside their existing data collection forms. They were observed in the field (by SI), who monitored participants' use of the form, the time taken for completion and suggestions for improvement. He later added his own suggestions, based on an understanding of the context in which it was used. Participants were invited to a final workshop following the trial period to provide feedback of their experiences of completing the reporting form.

Following the informal testing of the form in the field, we conducted formal pretesting in Kampala. Pretesting involved testing each section of the form for comprehension (interpretation of pictures, text and ideas), and for usability (correct completion of the fields). First, we pretested an active reporting form, adapted from the draft passive form, with university graduates acting as potential field workers. We then applied relevant revisions to the passive form and pretested this form with graduates and CMDs.

Pretesting involved a series of day-long sessions attended by around 10 graduates or CMDs (‘users’) and facilitated by a trained pretesting team. In each session, users were given an introduction to pharmacovigilance and the purpose of the pretesting, before participating in training in how to use the draft reporting form. Each user then completed the form by interviewing a member of the pretesting team acting as the patient or caregiver. In each round, users tested the form in up to three pre-prepared clinical scenarios. During the role play, the pretester observed how the user completed the form and any problems that emerged. On completing the form and ending the role play, the pretester asked the user for their feedback and made notes on comprehension and use of the form. Pretesting workshops were held in English, although for the passive form pretesting with CMDs, members of the research team were able to translate to the local language where necessary.

Reviews were undertaken after each pretesting session. A quantitative analysis calculated the proportion of the entries on the form that were ‘correct’ when compared with a pre-completed version of the form. Comprehension was assessed by looking at those sections of the form that were less well completed and addressing misunderstandings about how to complete those sections. The forms were updated based on feedback from participants at the end of each day, and the pretesting cycle continued. We aimed to achieve 80–90% understanding of the reporting form.

### Ethics Statement

All participants were given information about the project and were asked to give verbal consent to participate. Ethics approval for this work was received from the Research Ethics Committees of Uganda National Council of Science and Technology (Reference number HS433), Makerere University (Reference number 2008-037), London School of Hygiene and Tropical Medicine (application 5241) and the Liverpool School of Tropical Medicine (protocol 09.56).

## Results

### Review of existing forms

21 participants demonstrated, discussed and evaluated existing pharmacovigilance tools with the project team. The review revealed challenges completing all available AE forms. In many completed forms, we observed inconsistency in detail about the event and the patient, limiting the scope of AE assessment. The key problems we identified with completion of the forms are described in Supporting Information S1. In addition to challenges specific to the format of forms, our analysis highlighted challenges with the wider process of AE reporting. These included identifying a case as relevant to report, eliciting relevant information from patients, interpreting patient explanations, and recording information on the reporting form.

#### Focus Group Discussion

Ten CMDs, all active in pharmacovigilance, and nine health workers with a range of experience and qualifications and no prior AE reporting experience, participated in the two workshops with focus group discussions to review existing forms, and then subsequently in the form development workshops.

In their focus group discussion, the challenges of interpreting and recording the patient's story were described. CMDs revealed specific logistical challenges of the existing forms:

*‘We have to jump from the first column to the fourth column and then to the sixth and back.’*

*‘Types of drug are difficult to record-some people don't know what has been taken.’*

The CMDs described how patients presented information to them in terms of the trajectory of the illness, including the timeline of the symptoms and treatment, which were often described in relation to other non-medical events.

*‘This is what we have been complaining of. It [the form] doesn't bring out what we get from our clients. If you are telling someone a story and use that form, they will not get what you are saying … We don't move with our forms – we move with small books where we write, then after we come back and fill in.’*

The need to capture more of the complexity around patient narratives was identified by CMDs as central to a useful reporting form.

Health workers reported that they generally believed side-effects were already well known by the drug manufacturers who list them on leaflets and packaging. Serious AEs were also identified, such as optic atrophy and severe anaemia, but these were still conceptualised as expected side-effects that needed treatment or advice. Health workers and CMDs described challenges of motivating patients to report information about AEs. CMDs identified changes in community involvement over time: lack of feedback from reporting of previous events had made them less likely to report current events. Health workers reported that eliciting accurate information from patients was challenging, particularly regarding what medicines they had taken, especially herbs. Although health workers reported probing for other drugs, patients were reluctant to tell them because of ‘stigma’ and ‘fear’ because ‘*they think we will react to them*.’ A clear need was expressed for education of both patients and health workers about the reasons for, relevance and value of reporting.

### Form development

**Figure 2:**
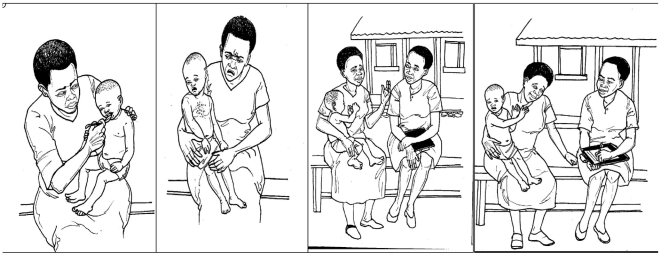
Pictorial strip first draft, demonstrating reporting of a child's adverse event.

We evaluated our review of existing forms in the light of communication and visual perception principles and previous field experience. We developed the idea of a diary format to represent chronological events, and a pictorial strip to show and explain the purpose of reporting AEs, and the need for cooperation between the respondent and reporter. Two series of pictures were developed by the artist with guidance from the communication specialist; one for adults and one for children, using line drawings. Figure 2 shows the draft of the pictorial strip for reporting adverse events in children.

### Documenting the patient's story chronologically

In their original drafts of the form, CMDs followed formats they were familiar with, writing a series of questions in columns. However, in role-plays, participants identified that this style had limitations in capturing the necessary data, especially regarding the relationship between dosage timing and symptoms of emerging events. When the diary format was introduced, the participants responded positively as it enabled them to *‘bring out the whole story.’* In the second workshop, the health workers built on the diary format idea, suggesting additional fields for inclusion including specifics of patient information.

Participants decided that the core of the patient story should be reported in the diary, helping the patient to follow what they are recording, and prompting the patient to think chronologically. Participants also recognised the need for additional fields to document further details about the reported information and the diary was coupled with a more traditional column-based section for follow-on questions.

#### Communicating the purpose of the form

The importance of cooperation between the person completing the form and the respondent was emphasized. Presented with the draft pictorial strips, CMDs and health workers discussed and tested their contents and use, and suggested revisions to the pictures. They also suggested adding text to remind the user to show and explain the pictures to the patient. The artist undertook several revisions of the pictorial strips before arriving at the final version for pretesting.

#### Review of forms after practice

Following practice with the draft form at home, the CMDs shared their experiences and made suggestions for improvements. The diary format was well accepted, as was the storyboard, which appeared to achieve its objectives of improving patient motivation to report and cooperation with the reporter:

*‘People can understand this picture… As I was filling the form they were experiencing what I was doing’*

*‘From the pictures I have seen that the CMD must be humble and listening and you show the information, what you write, what he's telling you, you must be aware. And it says why you are visiting, why it is needed to report.’*

Generally, forms were completed as intended, although there was some confusion over where to record data on the diary, and the wording of some structured questions. Revisions were made, and an example on a template form was created to help reporters to remember how to complete the diary section.

### Pretesting

#### Practice and observation in the field

Over two weeks, 13 CMDs and one health worker were observed completing the form. Overall, the concepts appeared to be well understood; however, the diary format and tables required explanation to the respondent and further explanation to reporters. The language used in the form (English) was not used in the interaction between the reporter and the respondent. Adjustments were made to the draft form, with the intention to translate final versions of the form for local use where necessary.

The pictorial strips was sometimes overlooked, particularly in literate patients,

*‘The pictures are not needed if the person is learned; they understand why we are here’.*

However, both respondents and CMDs were keen that the pictures remained, and interpreted them in the intended way whenever questioned. When the observer asked the CMD to begin with the storyboard when interviewing a respondent who appeared literate, the session was a more interactive engagement between interviewer and respondent, providing good reason for the pictorial strips to be retained with minor adaptations.

The passive draft form was adapted for active data collection. The two forms were similar except on the active form additional space for recording baseline information including prescription and laboratory data was included.

#### Formal pretesting in Kampala

**Figure 3:**
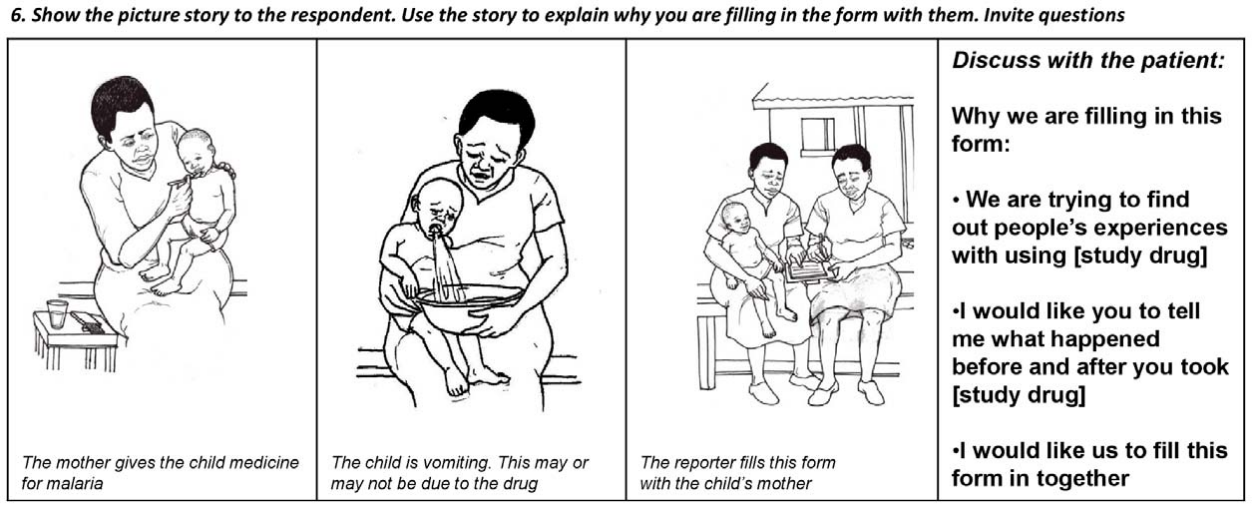
Pictorial strip after workshops and pretesting for active reporting.

Formal pretesting of the forms was carried out over 8 days in Kampala. Five rounds of pretesting were completed for the active reporting form, with 50 university graduates, using three different clinical scenarios (detailed in Supporting Information S2). The median score for the form was high, with the greatest challenges found in the diary section. As the pretesting progressed, changes included improving training in the use of the diary, revising the question wording, and changing the storyboard picture from rash to vomiting which was considered a better choice for patient understanding (Figure 3). The change of picture was specific to active forms: users felt the vomiting image encouraged them to report any symptom, rather than symptoms traditionally associated with drug reactions, such as rash. The median total score improved across the different sections of the reporting form from 79% in the first round to 92% in the final round. Scores are presented in Table 1.

**Table 1:** Use of active reporting form over five pretesting and revision sessions.

Day	No of participants	Role-play scenario	Median diary score (%)	Median total Score (%)
***1***	*9*	*A*	*61*	*79*
***2***	*10*	*A*	*70*	*75*
	*6*	*B*	*66*	*58*
***3***	*10*	*A*	*68*	*85*
***4***	*11*	*B*	*86*	*86*
	*3*	*C*	*96*	*98*
***5***	*8*	*A*	*77*	*92*
	*9*	*B*	*75*	*87*
	*8*	*C*	*77*	*89*

**Figure 4:**
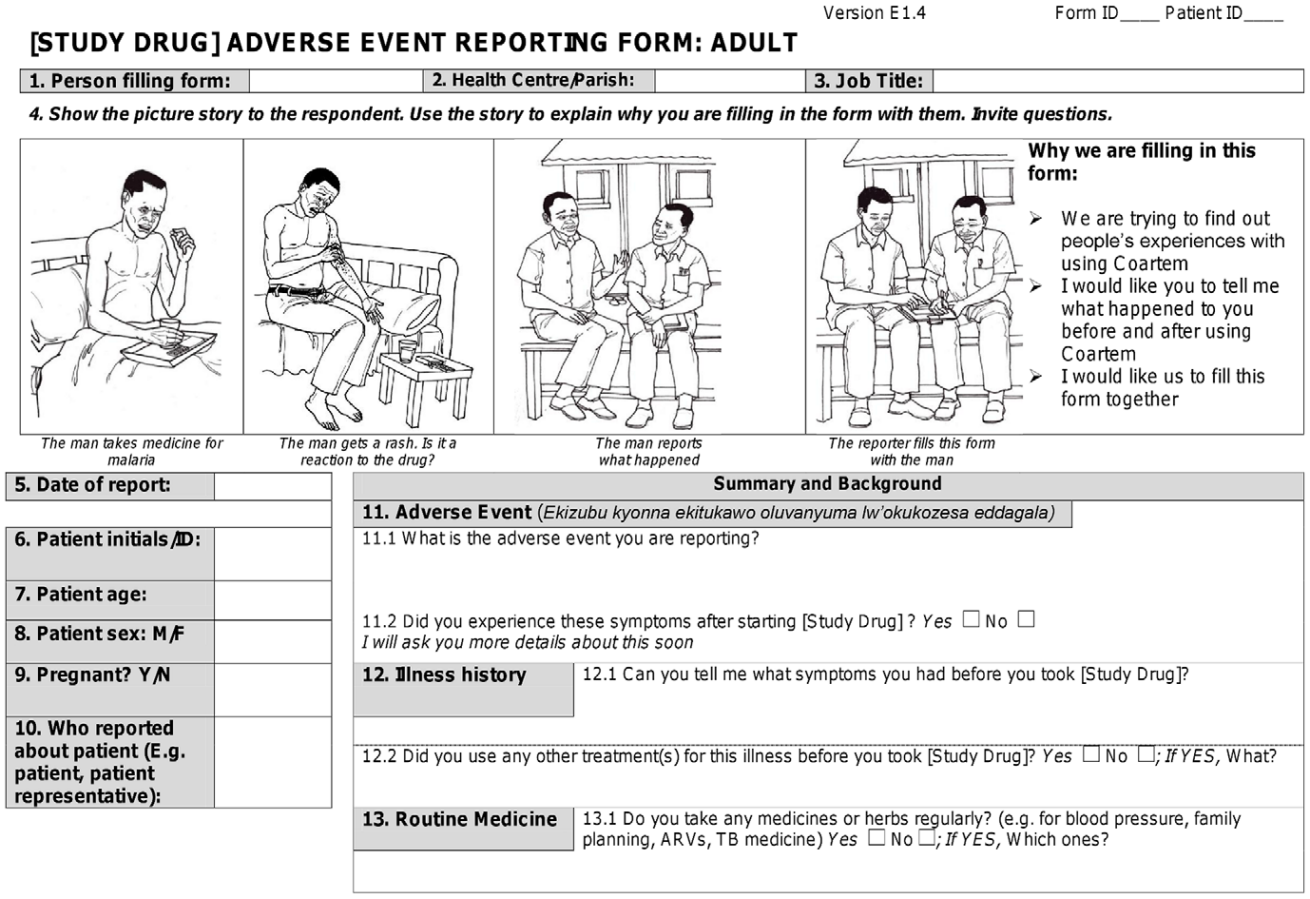
Final draft of passive reporting form for adult patient (Page 1).

**Figure 5:**
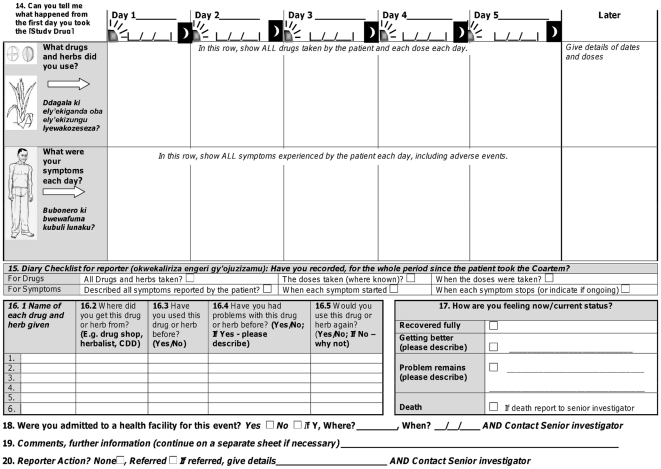
Final draft of passive reporting form for adult patient (Page 2).

Three rounds of pretesting of the passive reporting form were completed with 9 graduates and 17 CMD users, using three different clinical scenarios (Supporting Information S3). Users completed these passive forms well, with high median scores for both graduates and CMDs (Table 2). We made minor revisions to the form in each round of pretesting, for example changing ‘antimalarial’ to recognised local brand names, and providing specific training on parts of the form, such as the use of arrows to indicate the duration of an event. Overall, we received positive feedback on the use of the pictures and users stated that the form could be used easily with practice. The final draft of the passive AE reporting form is shown in Figure 4 and Figure 5. Completed versions of the active and passive forms can be found in Supporting Information S4 and Supporting Information S5.

**Table 2:** Use of passive reporting form over three pretesting and revision sessions.

*Day*	*Type of Participants*	*Number of participants*	*Scenario*	*Median Total Score (%)*
***1***	*Graduates*	*8*	*A*	*85*
		*3*	*B*	*71*
		*9*	*C*	*77*
***2***	*CMDs*	*6*	*C*	*80*
***3***	*CMDs*	*6*	*A*	*86*
		*6*	*B*	*81*

## Discussion

Expanding pharmacovigilance programmes beyond the formal public health sector presents an opportunity to improve the coverage of collection of drug safety data. Using a participatory research approach, we developed and pretested user-friendly AE reporting forms designed to be used by lower-level health workers and non-clinicians, and achieved high reporting accuracy: over 80% of the sections were completed correctly in role-play scenarios.

We found that existing forms were often poorly completed, even by clinicians, in line with previous studies [3], [6]. A major problem with existing forms was their ‘black boxing’ of the processes of translating and transcribing experiences to recorded reports: the realities of patients' illness events and the elicitation and documentation processes did not fit well onto the reporting forms that required pre-processed ‘data.’ Consistent with findings elsewhere [7], [8], [15], participants described difficulties for patients to report ‘relevant’ information, for reporters to filter this into ‘relevant’ data and for those receiving reports to attribute causality in a relative vacuum of information. The design of our reporting form took into account the difficulty of establishing relevance for data reported and attempted to tackle this in several ways. For the patient, the storyboard helps to set the scene for reporting and the importance of establishing a partnership with the reporter. The pictures show an equal rather than hierarchical relationship between the patient and the reporter by showing them both looking at the form and sharing information, known to be important if information is to be shared freely [17]. This reflects that the diary is intended to be completed by the reporter and respondent together, to reduce the power differential assumed in paternalistic health worker-patient relationships and promote equal responsibility, trust and consensus in the decision [18], in this case what to record on the form. For the reporter, the aim of the form was shifted from documentation of an adverse drug reaction to documentation of an illness episode, removing responsibility for establishing causality. The design of the form helps to do this by prompting for all symptoms and medical events over the course of the illness, including pre-existing symptom and medications, and the importance was reinforced in the preparatory training for reporters. For the interpreter, far greater information is available to inform causality assessment, particularly regarding the chronology of events. Establishing clear chronology also assists in differentiating pre-existing symptoms from those developing after the suspected drug is taken and establishing if the reported symptoms resolve on discontinuation of the medicine or recur following recommencement of the medicine.

The participatory approach used in this research had a significant impact on the format and content of the reporting form developed. Participants influenced the design of pictures and content for fields, and continuous rounds of testing enabled us to identify important changes that enabled improved understanding and completion of the forms. This follows other similar activities that have developed successful community relevant strategies for malaria and for onchocerciasis in Africa [16], [19], [20].

Our results suggest that non-clinicians are able to record good quality drug safety data and could make important contributions to pharmacovigilance programmes. This challenges current WHO guidance that is cautious in its recommendation, ‘because of their varying degrees of literacy [they] cannot act as reporters, but should play an important role in referring patients to health facilities to report reactions’ [21]. The approach we have taken, asking the reporter to record all symptom and drug events on the form, leaving interpretation of causality to those receiving the data, could address these concerns, particularly the observation that AE reports are ‘the product of the experience and diagnostic logic of the reporter’ [22] . The development of these forms also supports the recommendation from the WHO-MMV pharmacovigilance consultation for the Affordable Medicines Facility - malaria that simplified forms should be created for community health workers and medicines vendors [23]. The use of low-educated but trained workers to collect such information could provide a middle-ground between clinician reports and direct reports from patients and parents [24]–[26]. The collaboration with health personnel at such levels is also important to strengthen sustainability, as lower-level personnel tend to be more stable in the community.

There are limitations to our approach. Pharmacovigilance programmes are complex and success relies on many factors beyond reporting forms [27]. Further evidence is needed to support the integration of community health workers in pharmacovigilance activities at scale in routine programmes, especially in relation to the meaning this role accords and sustainability of motivation for volunteers in communities [28], as well as the need for and cost of training and supervision for such programmes [29], [30]. User-friendly reporting forms with evidence of usability by lower-level health workers and non-clinicians therefore addresses just one gap for implementation.

The current design of the form has a short diary period, with the focus on immediate and short time to onset events. Recall is usually better over this short time period [31], although it would be useful to assess whether chronology of events is accurately recalled and captured with this form. Capture of delayed onset events might be facilitated by an extension of the diary to a period of weeks, and space for detailing narratives, but recall issues for respondents and the level of literacy and pharmacovigilance expertise required by reporters to unpick increasing numbers of confounding factors means that such cases are best reserved for active follow-up by specialised health workers. Due to practical restrictions in the number of fields, the forms do not capture data on resolved symptoms or completed medicines prior to the illness episode, which could limit interpretation of data. In addition, support would be needed for these forms to be used effectively, particularly training of reporters in completion of the diary component, and for the principles behind the storyboard. Scale-up of the form would first require its evaluation under different conditions. The form reported here is demonstrated in our particular project population, has a focus on malaria and artemether-lumefantrine, and used pretesting scenarios that may not reflect the complexity of real life situations. Evaluation of the forms in other settings, under routine reporting conditions, and in comparison with existing forms would provide a degree of generalisability and validity for antimalarial-specific pharmacovigilance. Adaptation of the form, or its central components of the storyboard and diary concept, with encouragement of the reporter to ‘tell the patient's story’, could be tested for drugs to treat other diseases.

### Conclusions

We took a participatory approach to create novel and effective reporting forms for collecting much needed pharmacovigilance data in resource-limited settings. The forms have been developed and tested with a focus on antimalarials, in test scenarios in Uganda, with planned field evaluations in programmatic settings. The forms or their components could be adapted and tested for other medicines, to encourage a unified patient-focussed approach to pharmacovigilance reporting.

## Supporting Information

Supporting Information S1 **Problems identified in review of existing forms by end-users, experts and the project team.**
(PDF)

Supporting Information S2 **Active pretesting scenarios.**
(PDF)

Supporting Information S3 **Passive pretesting scenarios.**
(PDF)

Supporting Information S4 **Completed active reporting form.**
(TIF)

Supporting Information S5 **Completed passive reporting form.**
(TIF)
