# Supplementary material for: Designing Adverse Event Forms for Real-World Reporting: Participatory Research in Uganda
Source: PLoS One. 2012 Mar 29;7(3):e32704. doi: 10.1371/journal.pone.0032704 (PMC3315549; doi:10.1371/journal.pone.0032704)
Supplement: Supporting Information S1 — Problems identified in review of existing forms by end-users, experts and the project team. (PDF) [file pone.0032704.s001.pdf]

Supporting Information S1:

Problems identified in review of existing forms by end-users, experts and the project team

Unclear fields

- Some fields were misused or unused on all forms, by reporters of all cadres. E.g. 'duration of drug' completed as the intended duration rather than duration actually taken.
- Free-text sections lacked guidance on use.
- Field headings were often broad, lacking explicit instructions for inserting or interpreting contents. E.g. 'other drugs used' field included records of treatment at the time of the report, treatment at the time of the event and co-medication but these data could not be differentiated.

Language

- Some of the wording was difficult for reporters to understand, for example, 'anaphylaxis.'
- The use of English rather than local languages may have inhibited understanding and appropriate use of the reporting forms.

Insufficient space

- Forms provided insufficient space for detailing the AE described, resulting in less detailed accounts or forcing the reporter to continue outside of the allocated space.

Leading requests

- In forms with tick boxes for AEs, these were restricted to 9 boxes and one 'other.' We observed that check boxes could limit how well an event was described. 'Skin reaction', for example, could encompass several conditions of differing origins, and be localised or generalised. Details of which, if available, would be important for the assessment of the event.
- In free-text fields for event details, a narrow range of symptoms were reported most frequently. Informants remarked that previous pharmacovigilance training had focused on well-known events that were interpreted as 'relevant' to report, for particular drugs.

Unknown requests

- Reporters, particularly CMDs, struggled to complete certain fields that required information they did not have access to, such as 'manufacturing date' and 'batch number.'
- Leaving fields incomplete was uncomfortable for some reporters, for example informants noted that they felt obliged to complete the field 'date reaction finished' even if the reaction was continuing.

Matching the form with reality

- Forms that asked for only one 'suspected drug' required the reporter to make such a judgement and did not account for the reality of multiple concomitant medicines being used.
- Multiple symptoms at different time points, with some getting better or worse, could not be captured on the forms but were considered important information by informants.
- There was no space for herbal medicines to be noted.

Missing fields relevant for case review

- Forms did not allow space for symptoms that prompted treatment, prior to the AE. Informants considered this important for attribution of causality.
- Informants were concerned that forms were not designed to capture an individual's history, including whether the drug had been taken previously, whether the reaction had occurred previously, whether the AE stopped when a specific drug was stopped or restarted when the drug was restarted.
