# Supplementary material for: Designing Adverse Event Forms for Real-World Reporting: Participatory Research in Uganda
Source: PLoS One. 2012 Mar 29;7(3):e32704. doi: 10.1371/journal.pone.0032704 (PMC3315549; doi:10.1371/journal.pone.0032704)
Supplement: Supporting Information S2 — Active pretesting scenarios. (PDF) [file pone.0032704.s002.pdf]

Design of an adverse event monitoring form for non-clinician reporters: participatory research in Uganda

Supporting Information S2: Pretesting Scenarios for Testing of Active AE (adverse event) monitoring form for non-clinician reporters

Scenario A (For Fieldworker)

- You go to the **health centre** which is part of your study.
- There, you look at the register for patients who have been prescribed Coartem.
- This is the record you see for M Ibyara (Patient ID 0019)

Date	Patient Name	ID	Symptoms	Diagnosis	Test results	Treatment
04/07/09 Saturday	M Ibyara	0019	Vomiting, fever, headache	Malaria	RDT +ve	Coartem 4x2 (3 days)<br>Domperidone – 20mg once<br>Panadol – 2 tablets ONCE

- You are visiting her at home on day 3 after being prescribed Coartem: DD-MMM-YY ____day. You speak to the patient

Scenario A for Patient

Maureen, 37 year old female, Weight 55kg, Height 158cm, she is not pregnant
No other medicines

____day (4 days before pre-test)

Maureen was beginning to feel unwell with a **headache and fever**. She went to the local healer and took a **herbal drink** – she does not know what it was. The headache and fever continued.

___day (3 days before pre-test)

Maureen took the herbal drink again in the morning but was getting worse and vomited twice in the afternoon.

____day (day before yesterday, 2 days before pre-test)

Maureen visited the **health centre** because she was feeling very unwell with **fever, 39 degrees, headache and vomited twice on ___day morning**. She received **one dose of anti –nausea medicine (domperidone) on that day and one dose (two tablets) of panadol at the health centre**. She was prescribed **coartem and took the first dose (4 tablets) in the afternoon and the second (4 tablets) that evening**.

___day (yesterday)

On ___day, she **took coartem (4 tabs) in the morning** but was still feeling ill **with headache and vomiting** (twice). In the afternoon she had an **episode of blurred vision. For about half an hour it felt like she was seeing everything through smoke. She took coartem again (4 tabs) in the evening**. Her **fever had improved, but was still there.**

___day (Today)
Maureen took **4 Coartem tablets in the morning**. She **still had a headache and the vomiting she had had for the previous three days was also much worse, 5 times this morning**. She has **no more fever**. She is experiencing **extreme sleepiness**.

Notes
She has not taken Coartem before but she would take it again if the doctor told her to
She has taken panadol before but not the anti-nausea drug given at the Health centre, but she would use them both again
She is worried about her health.

Scenario B (For Fieldworker)
You go to the health centre which is part of your study.
There, you look at the register for patients who have been prescribed Coartem.
This is the record you see for S Francis (Patient ID 0016)

Date	Patient Name	ID	Symptoms	Diagnosis	Test results	Treatment
03/07/09 Friday	S Francis	0015	Fever, Vomiting	Malaria	RDT +ve	Coartem 1x2 (3 days)<br>Panadol syrup 5ml x4 (2 days)<br>ORS 1 x2 (3 days)

You are visiting him at home on day 4 after being prescribed Coartem: DD-MMM-YY ____day. You speak to his mother.

Scenario B (For patient's mother)

Patient
Samuel Francis, male, 18 months old, weight 10kg, height 80cm
He does not take any other medicines

History
______day (3 days before pre-test)
- The episode of malaria started with **fever (39.5 degrees)** and **diarrhoea** four days ago (on Friday). The mother gave the child one **panadol** tablet she had in her home and visited the health centre.
- He was given **Coartem and ORS** at the health centre in the afternoon, but his mother did not buy the panadol syrup prescribed
- Samuel was given **one coartem tablet again before he went to bed**. And **one ORS sachet at night**.

________day (day before yesterday, 2 days before pre-test)

- When Samuel woke on this **morning he had a rash on his tummy, it was made up of tiny** dots. **His fever was much better but he was still a little hot**. He had **diarrhoea** again.
- He was given another **ORS sachet in the morning**
- His mother gave **one more dose of Coartem in the morning**
- By the late afternoon the rash had spread all over his tummy, back and chest and was itching Samuel. He had no more diarrhoea and the fever was better so his mother did not give any more ORS and she was worried that the Coartem had caused the rash so she did not give him any more.

_____day (Yesterday)

- Samuel still had no fever and no diarrhoea
- Samuel's **rash was still itchy** but was not as red as before. It still **covered his chest and back**
- Samuel's mother visited the drug shop and she bought some cream for the rash (don't know name)
- She put the **cream on Samuel in the afternoon**.

_____day (Today)

- The **rash is now better but not gone** completely – it **does not itch** any more
- He has no other symptoms
- Samuel's mother applied the cream this morning

Notes

- He has taken ORS and panadol before without problems and would use again
- He had Coartem before but had no problems before
- She would not use coartem again, would use cream again

Scenario C (For Fieldworker)

You go to the drug shop which is part of your study.
There, you look at the register for patients who have been prescribed Coartem.
This is the record you see for J Maliga (Patient ID 0012)

Date	Patient Name	ID	Symptoms	Diagnosis	RDT Test result	Treatment
04/07/09 Saturday	Joseph Maliga	0012	Fever, cough	Malaria	Positive	Coartem 4x2 (3 days)<br>Panadol 2x4 (3 days)

You are visiting him at home on day 3 after being prescribed coartem: DD-MMM-YY ______day. You speak to the patient.

Scenario C (For patient)

Patient

Joseph Maliga, male, 48 year old, 175cm tall, weighs 70kg

- TB patient on long-term medication for **TB** – doesn't know names of drugs

History

_______day (4 days before pre-test)

- Illness episode started with **fever**, **persistent cough** and **joint pain**
- Took **panadol** and **SP** on the same day at a drug shop

_______day (3 days before pre-test)

- Fever persisted, and I kept coughing and the joint pain in my knees continued

______day (day before yesterday, 2 days before pre-test)

- Fever persisted, and I kept coughing and the joint pain in my knees continued
- Visited another drug shop. They did a **new-style test** and told me I had **malaria** at the drug shop. They gave me **Coartem** and some more **panadol**.
- I took **four Coartem tablets this afternoon** and another **four Coartem that evening**, and **took 2 panadol tablets at the same time in the evening**. I still had fever, pain and cough.
- Took TB Medicine (2 capsules) as normal

____day (Yesterday)

- I still felt ill so took **Coartem dose(4 tablets) three times throughout the day (dawn, midday, dusk)**
- In the evening the fever and pain had gone. But the cough remained.
- Took TB Medicine (2 capsules) as normal

_____day (Today)

- This morning I have some **difficulty breathing, feel very short of breath** even when resting
- I **have not taken any Coartem this morning**. I am thinking of going to the health centre.
- Took TB Medicine (2 capsules) as normal

Notes

- Previously taken panadol and SP before but not Coartem
- Never had any problem with panadol or SP and would take them both again
- Had a rash when first started TB treatment
- Would not take the Coartem again
- Now, problem continues
