# Supplementary material for: Designing Adverse Event Forms for Real-World Reporting: Participatory Research in Uganda
Source: PLoS One. 2012 Mar 29;7(3):e32704. doi: 10.1371/journal.pone.0032704 (PMC3315549; doi:10.1371/journal.pone.0032704)
Supplement: Supporting Information S3 — Passive pretesting scenarios. (PDF) [file pone.0032704.s003.pdf]

Design of an adverse event monitoring form for non-clinician reporters: participatory research in Uganda

Supporting Information S3: Pretesting Scenarios for Testing of Passive AE (adverse event) monitoring form for non-clinician reporters

Scenario A

Patient

Maureen, aged 37 years old

- Maureen has come to see you
- She does not take any regular medicines

Maureen has heard you are collecting information on problems people are having with Coartem and she wants to tell you about something that happened to her last week.

Event the patient is reporting

Had blurred vision after taking Coartem. It felt like she was seeing everything through smoke. It happened about 3 hours after she took the last dose of the medicine last Friday.

History and other details

Tuesday (previous week) (DD-MMM-YY)

Maureen was unwell last week. She had fever and headache last Tuesday and first went to the drug shop and took some powder they gave her (unsure of name)**.**

Wednesday (previous week) (DD-MMM-YY)

The fever did not improve and so she went to the health centre on Wednesday - she still had fever 'on and off' and a severe headache. They said she had malaria and was prescribed coartem and panadol. She took 2 coartem the morning and 2 panadol. In the evening she took the same drugs and doses again. By the evening the fever had gone but she still had a headache and her eyes hurt when she opened them (she didn't like bright light'). She went to bed early.

LAST Thursday, the headache was still there but she didn't have the eye pain. She took half the coartem (2 in morning and 2 in evening) because the fever had gone and she thought that would be enough. She didn't take panadol. The headache had gone by evening.

LAST Friday, she decided to take the full dose of coartem in the morning (4 tablets) and 3 hours later had the episode of blurred vision. It lasted for about half an hour.

Since then...

She didn't take any more Coartem tablets and is fine now

Maureen has never taken Coartem before

- She would not use this again

- He has had panadol without problems

Scenario B

Patient:

Fatima Juma, female, age 24 years

- She is pregnant (24 weeks)
- She does not have any other medical conditions

Event she is reporting

Yesterday afternoon (_____**DAY**) **her left side went numb**: she could not move her left arm or hand, or the left side of her face, 3 hours after taking first dose of Coartem. The symptoms continued until the evening when she went to bed. When she woke up this morning, the symptoms had resolved. Now she feels fine

History and other details

_____day (two days before pre-test day)

She started feeling ill on ____day morning with fever and vomiting. Fatima took a local herb she got from her grandmother: the neem tree, mixed into a hot drink. The fever and vomiting continued and she went to the health centre in the afternoon. She was prescribed co-artem but did not buy on Tuesday.

_____day (Yesterday)

Bought Coartem in the morning at the drug shop and took 4 tablets.

- Almost immediately (3 hours) after taking the first 4 tablets of Coartem she lost movement in her left arm and left hand and the left side of her face.
- Fever and vomiting stopped by evening

_____(Today)

Today she feels OK. Has not taken any more medicines but has returned to the herbalist and now has a herbal drink with ekikaka. She has taken one cup this morning.

- She has never taken Coartem before
- She has used the neem tree before without problems
- She stopped taking the drug
- She would not take the drug again

Scenario C

Patient

Susan, age 3 years old

- The patient's mother has brought her to you
- She does not take any regular medicines

Event the mother is reporting

Susan has had **nightmares** for the past two days and is very **fatigued**

History and other details

_____day (three days before pre-test day)

- Susan had **fever (38.5 degrees) and aches and pain**s and was not playing three days ago on ___day**.**
- Her mother took her to the CDD that afternoon who gave her one dose of coartem (one tablet) straightaway
- Susan still had fever in the evening and was given another coartem tablet late in the evening on ___day.
- She slept for only 2 hours on ___day night

_____day (two days before pre-test day)

- She was fatigued all of ___day and would not play happily with her sisters, but the fever seemed to be better. She was given one tablet of Coartem in the morning and one in the evening as the CDD said.
- She still had difficulty sleeping on ___day evening and woke up many times in the night with nightmares. Still had fever on ____day, but no more joint pain by the afternoon

_____day (Yesterday)

- The fever had gone by the morning. She seemed dizzy on ______day when she was with her sisters, and fell over on this morning, but took Coartem in the morning and the evening.
- She had nightmares again yesterday and hardly slept.
- She finished the Coartem course

______day (Today)

- Has had no more drugs today

- Susan has never taken Coartem before
- Her mother would not use this again
